# Mutational Profiles of Cutaneous Squamous Cell Carcinomas with Different Patterns of Clinical Aggression from Head and Neck Regions

**DOI:** 10.3390/cancers16111956

**Published:** 2024-05-22

**Authors:** Maria Colombino, Giuseppe Palmieri, Manuela Rodio, Matilde Tettamanzi, Silvia Rampazzo, Raffaello Margani, Emilio Trignano, Antonio Cossu, Maria Antonietta Fedeli, Giovanni Maria Fadda, Corrado Rubino

**Affiliations:** 1 Institute of Genetic and Biomedical Research (IRGB), National Research Council (CNR), 07100 Sassari, Italy; maria.colombino@cnr.it; 2 Immuno-Oncology & Targeted Cancer Biotherapies, Unit of Cancer Genetics, Institute of Genetic and Biomolecular Research, National Research Council (CNR), University of Sassari, 07100 Sassari, Italy; 3 Plastic Surgery Unit, University Hospital Trust of Sassari, 07100 Sassari, Italy; m.rodio@studenti.uniss.it (M.R.); m.tettamanzi@studenti.uniss.it (M.T.); s.rampazzo93@studenti.uniss.it (S.R.); r.margani@studenti.uniss.it (R.M.); emilio.trignano@aouss.it (E.T.); corubino@uniss.it (C.R.); 4 Plastic, Reconstructive and Aesthetic Surgery Training Program, University of Sassari, 07100 Sassari, Italy; 5 Department of Medicine, Surgery and Pharmacy, University of Sassari, 07100 Sassari, Italy; 6 Unit of Anatomic Pathology and Histology, University Hospital of Sassari (A.O.U. SS), Via Matteotti 60, 07100 Sassari, Italy; antonio.cossu@aouss.it (A.C.); mariantonietta.fedeli@aousassari.it (M.A.F.); 7 Oncologia Medica, University Hospital of Sassari (A.O.U. SS), 07100 Sassari, Italy; giovanni.fadda@aouss.it

**Keywords:** non-melanoma skin cancer, cutaneous squamous cell carcinoma (cSCC), recurrent cSCC, next-generation sequencing (NGS), mutation profile, immunogenicity

## Abstract

**Simple Summary:**

Cutaneous squamous cell carcinoma (cSCC) ranks among the malignancies with the most pronounced mutation burdens. Understanding its genetic aspects is crucial for improving patient care. This retrospective case-control study compares the molecular profiles of cSCCs in the head and neck regions prone to local recurrence against those without relapse, aiming to identify predictive markers for prognosis and therapy. Using a next-generation sequencing (NGS) array, histological samples from cSCC patients were analyzed, highlighting the most frequently mutated genes in cSCC. Controls displayed a higher mutation count, thus potentially enhancing tumor antigen presentation for the immune system. This immune response might prevent future recurrences, emphasizing the immune system’s role in cSCC. The study underscores the importance of novel therapies that leverage the immune system.

**Abstract:**

Cutaneous squamous cell carcinoma is a prevalent malignancy with a rising incidence and a notably high mutational load. Exploring the genetic nuances of cSCC and investigating molecular approaches stands as a potential avenue for improving outcomes in high-risk patients. This retrospective case-control study involved two cohorts, one of 14 patients (the “discovery cohort”) and the other of 12 patients (the “validation cohort”), with cSCC located in the head/neck anatomical region and diagnosed at the pT2 stage. Overall, cases developed early local relapses of the disease, whereas controls never relapsed during the entire follow-up period. A next-generation sequencing (NGS) approach conducted on histological samples revealed that TP53 and CDKN2A were the most frequently mutated genes in our series. No specific mutations were identified as potential prognostic or therapeutic targets. Controls exhibited a tendency toward a higher mutational rate compared to cases. It is possible that an increased number of mutations could prompt the cSCC to expose more antigens, becoming more immunogenic and facilitating recognition by the immune system. This could enhance and sustain the immunological response, potentially preventing future recurrences.

## 1. Introduction

Cutaneous squamous cell carcinoma (cSCC) represents the second most common type of non-melanoma skin cancer (NMSC), accounting for up to 25% of cutaneous malignancies, and is preceded in frequency only by basal cell carcinoma. Several studies state that its incidence is constantly increasing, in line with a growing elderly population and a more sensitive focus on skin cancer screening [1,2,3,4]. Caucasian individuals are more often affected, with the average onset after the mid-sixth decade of life and a higher incidence in males. Many risk factors have been described as associated with cSCC, such as ionizing radiation, chronic immunosuppression, HPV infections, and previous burns. Above all, exposure to ultraviolet radiation (UVR) and sunlight is the most recognized carcinogenic factor, having a direct correlation with the incidence of SCC [1]. This explains the greater onset of lesions in photo-exposed regions of the body, mainly the head and neck regions, in people with a clear or fair phototype and advanced age [5]. 

As in most cancers, the process of cSCC carcinogenesis passes through the progressive storage of subsequent mutations and/or genetic alterations, mainly in response to ultraviolet light damage. Overall, cSCC is characterized by a mutation burden much higher than that of many other common solid neoplasms, thus resulting one of the most highly mutated human cancers [6,7]. A deeper knowledge of the genetic and molecular basis of cSCC would be useful for developing better forms of treatment and may help improve outcomes for high-risk patients [8,9]. 

Actinic keratoses (AKs) are certified as cSCC premalignancies. Chronic UVR exposure drives epidermal keratinocyte to dysplasia, but identifying specific genomic alterations that promote progression from normal skin to actinic keratoses (AK) to cSCC is challenging, since very high levels of background mutations in keratinocytes are associated with UV damage. A model of cSCC development and progression has been proposed [10], highlighting that prolonged UVB exposure leads to mutational increase and carcinogenicity. More specifically, skin areas chronically exposed to UVB initially exhibit broad alterations on NOTCH 1-2-3 genes; these driver mutations, coupled with prolonged radiation exposure, contribute to the stepwise progression to AKs and in situ squamous cell carcinoma (SCCis) by acquiring more mutations affecting TP53, FAT1, KMT2C, PIK3CA, and CDKN2A. The transition to invasive cSCC is definitely accomplished with additional alterations in HRAS, ABI3BP, and IMPA1 genes, as well as with the impairment of the TGF-β signal pathway [11].

TP53 is the most commonly altered tumor suppressor gene in cSCC patients. Mutations occur in 54–95% of cases and are promoted at early stages of cSCC pathogenesis through sustained exposure to UVB radiation, which induces cytosine-to-thymine transition in dipyrimidinic sites (so-called “UV signature”), thus enabling cancer keratinocytes to clonally expand and preventing apoptosis [8]. UVR-induced C > T transitions have been identified as the predominant genetic drivers of the disease [11].

Other mutations occur in additional tumor suppressor genes, such as CDKN2A and NOTCH, and in oncogenes, such as RAS. CDKN2A normally codes for two proteins, p16INK4a and p14ARF, whose loss of function may lead to failure in cell cycle blocking and uncontrolled cell growth mediated by pRB and p53. NOTCH1 and NOTCH2 mutations are prevalent in over 75% of cases [12]; NOTCH 1 is a p53 target and its mutation results in an imbalance between cellular growth and differentiation, along with upregulation of the Wnt/beta-catenin pathway [8]. Among oncogenes, HRAS mutations are commonly present in cSCC (3–20%), rather than NRAS and KRAS [6]. All these mutations can act by altering many signaling pathways, such as NFkB, MAPK, and PI3K/AKT/mTOR, which in turn mediate the overexpression of EGFR [13]; this point in cSCC is commonly associated with a more aggressive phenotype and a poor prognosis for the patient but can, however, be targeted with specific drugs to inhibit cSCC progression [8].

TGF-β signal pathway disruption is associated with cSCC progression, acting as a key point for transition from AK to cSCC. Even epigenetic modifications may play a role in neoplastic transformation [14], such as the hypermethylation of gene promoters, as well as overexpression of microRNAs, which can act either as oncogenes or as tumor suppressor genes [15]. Overall, all these molecular alterations collectively culminate in constitutive promotion of cell division and proliferation, while inhibiting repair mechanisms and apoptosis.

In the head and neck regions, cSCC represents a common form of skin cancer, with high incidence in the anterior scalp region, forehead, and ears, posing a significant challenge in clinical management. Even when localized in this anatomical site, tumors can cause significant cosmetic alterations, significantly impacting patients’ quality of life. In addition to cosmetic implications, one of the main concerns in cSCCs of the head and neck is represented by the high rate of local recurrences. Despite apparently favorable conditions, local recurrence remains a significant challenge, with an incidence ranging from 5% to 10% in scalp tumors. The probability of local relapse is particularly difficult to define in cSCC lesions that are intermediate in size, corresponding to pathological stage T2 (pT2), which is characterized by tumors measuring between 2 cm and 4 cm with clear margins and a lack of risk factors associated with recurrence. The accurate prediction of local recurrence for such borderline cSCC lesions constitutes a challenge of considerable practical importance. Thus, understanding the basis of such relapses at the molecular level may be helpful to identify groups at greater risk for which to implement early diagnosis as well as design personalized therapies.

Our study is aimed at investigating further the genetic profiles of locally recurrent cSCCs collected in a hospital-based manner from the head and neck regions of patients. In particular, our retrospective case-control study aims to compare the mutation profiles of the main genes involved in skin cancer pathogenesis in a series of cSCC samples from two groups of patients with borderline stage pT2: cases that have exhibited a more aggressive clinical behavior in terms of local disease recurrence and controls that have not relapsed. Overall, the purpose was to identify a distinctive molecular signature acting as a potential molecular marker associated with prognosis prediction and, thus, to be able to stratify cSCC patients into subgroups at high or low risk of local recurrence.

## 2. Materials and Methods 

### 2.1. Samples

Patients diagnosed with cSCC in the head and neck regions and histologically proven stage pT2 (indicating lesions of dimensions > 2 cm and < 4 cm) with surgical margins free of disease who underwent surgery at the Plastic and Reconstructive Surgery Unit of the University of Sassari and subsequently developed disease recurrence in the same location were included in the study. In the enrollment of cSCC cases, particular attention was paid to exclude patients carrying any potential risk factor recognized as a confounding element, such as a chronic immunosuppressive condition or disease, previous treatment with radiotherapy for any cause, or a personal history of previous skin cancers or other malignancies. Our cSCC controls comprised patients who developed the tumor with the same features described for cSCC cases but remained free from relapse over time.

The study cohorts were drawn from a comprehensive database that included histological reports of all surgically treated cSCCs (*n* = 869) over nearly 7 years (from January 2016 to August 2022) in our Plastic and Reconstructive Surgery Unit of the University of Sassari. The database included patient details such as date of birth, age, gender, clinical status, tumor site and clinical features, date of surgery, surgical procedures with description of reconstructive choices, and histological report. The cohort of 14 patients (the “discovery cohort”; 7 cases and 7 controls, see above) was selected from the initial years of database construction (from January 2016 to December 2019) in order to ensure a longer follow-up period, providing a reliable time frame (>4 years) to assess the occurrence of any tumor relapse. To further assess the relationship between identified gene mutations and clinical behaviour, a “validation cohort” including 6 cases and 6 controls with the same disease characteristics previously described (head or neck region and pT2 stage) was used. The validation cohort included cSCC patients presenting a shorter period of follow-up (<3 years).

The study was performed in accordance with the principles of the declaration of Helsinki and was approved by the Research Ethics and Bioethics Committee of the National Research Council (CNR).

### 2.2. Mutation Analysis

For mutation analysis, genomic DNA was extracted from formalin-fixed paraffin-embedded (FFPE) tumor tissues of 5 µm thickness from both cases and controls using the GeneRead DNA FFPE tissue Kit (QIAGEN, Hilden, Germany). Using light microscopy, tissue sections underwent manual macrodissection in order to obtain tumor samples with at least 70% of neoplastic cells. Isolated genomic DNA and correspondent libraries were accurately quantified using fluorescence-based quantification methods such as Qubit dsDNA HS (High Sensitivity) Assay Kit (Life Technologies, Carlsbad, CA, USA).

Mutation screening was carried out through a next-generation sequencing (NGS) assay on the Ion GeneStudio S5 system, using the Ion AmpliSeq Cancer Hotspot Panel v2, which is now expanded to allow translational and disease researchers to fast-track oncology research that targets 50 oncogenes and tumor suppressor genes involved in tumorigenesis and includes 207 amplicons with a maximum length of 187 bp [16]. Data analysis was conducted by Ion Reporter Software, Version 5.10.2 (Thermofisher Scientific, Waltham, MA, USA), which is a bioinformatics tool designed to streamline data sequencing and facilitate both annotation and interpretation of gene variants.

Following the sequencing process, gene variants underwent careful classification into three distinct categories. Pathogenetic (P) or likely pathogenetic (LP) variants are variants characterized by their potential to induce structural and/or functional changes in the related proteins. These alterations may introduce early stop codons that can cause premature termination of protein synthesis (nonsense variants or frameshift), modify normal messenger RNA processing (splicing), and generally result in incomplete, unstable, and consequently functionally inactive protein products, or replace a single amino acid (missense variants) affecting protein function. In contrast, benign or probably benign variants, which are typically associated with a very low or negligible oncological risk, were not included in the final report. Variants of uncertain clinical significance (VUS) present the most challenging interpretation. Their impact on the corresponding protein functionality is unclear, and definitively linking the obtained genetic data with a specific elevated oncological risk is not feasible. VUS consist of nucleotide changes that necessitate further investigation to determine their clinical implications.

Considering the poor quality of the isolated DNA from archived tissue samples of our series, which thus affects the NGS library quality, coverage of 100 reads or greater (≥100) and a variant allele frequency (VAF) cutoff > 5% with at least 10 mutated alleles for each candidate amplicon were adopted for mutation selection criteria. Variants were screened against COSMIC v92 database (Catalogue of Somatic Mutations in Cancer; https://cancer.sanger.ac.uk/cosmic (accessed on 7 March 2024)) and VARSOME (https://varsome.com/ (accessed on 19 March 2024)) to identify already known somatic mutations and mutation types, respectively. 

The clinical significance of all identified variants was examined using the standards and guidelines for the interpretation of sequence variants recommended by the American College of Medical Genetics and Genomics’ (ACMG) Laboratory Quality Assurance Committee and the Association for Molecular Pathology (AMP).

For the statistical analysis aimed at assessing significant discrepancies in mutational patterns that may be associated with disease recurrence between cases and controls, the gene variants underwent a statistical comparison using Pearson’s chi-square test.

## 3. Results

According to the selection criteria described in Section 2, among the cSCC-affected individuals undergoing surgery over a 7 years period in our Plastic and Reconstructive Surgery Unit, we identified in the discovery cohort seven patients (cases) who developed the first local relapse of the disease within less than two years of follow-up and seven patients (controls) who never relapsed during the entire follow-up period (median, 4.9 years; range 4.1–6.3 years). The cSCC cases in the discovery cohort examined in our study consist of exclusively male individuals with a median age at surgery of 74 years (ranging from 54 to 86 years). In contrast, the cSCC control group included six male and one female subjects, with a median age at surgery of 77 years (ranging from 63 to 88 years). The overall average age of our study population was 75 (median 78, range 54–88), with nearly all series represented by male subjects (only one female included; 7% of the selected patients).

All primary cSCC lesions here investigated were located in the head or neck region and classified at disease stage pT2cN0cM0, categorizing them as high-risk lesions from the clinical point-of-view, according to SCC NCCN guidelines [17]. Specifically, among the entire series of 14 cSCCs analyzed, 6 (43%) were located on the cheeks, 4 (29%) on the scalp, 2 (14%) on the ears, and another 2 (14%) at the lip level.

Regarding the size of the lesions’ major axis, cases had an average of 2.8 cm, with a range between 2.2 cm and 3.6 cm, while controls had an average diameter of 2.6 cm, ranging from 2.1 cm to 4.5 cm. The overall average size of all excised lesions was 2.9 cm. 

In terms of the histological characterization of the tumors, a keratoblastic component was observed in 71% of cases and in 57% of controls, whereas in relapses only 29% exhibited this component. Ulceration was present in eight (57%) of both cases and controls; among patients with ulcerated cSCC, three (43%) presented disease recurrence. Finally, it was noted that 14% of cases presented solar keratosis at the site of excision, while 29% of the controls had solar dysplasia at the perilesional site.

**Figure 1:**
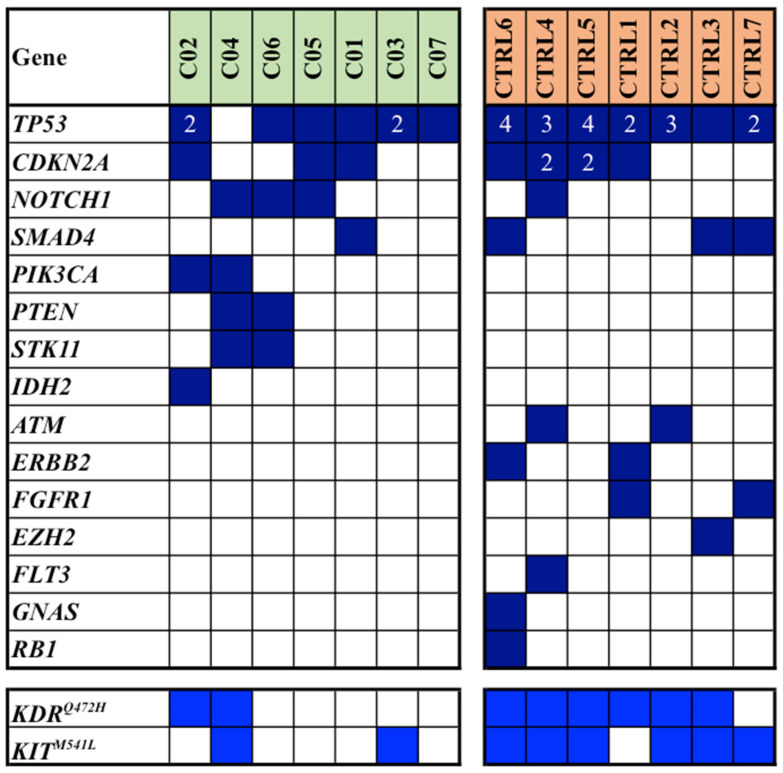
Spectrum and distribution of pathogenic/likely pathogenic mutations (dark blue squares) among cases (C) and controls (CTRL) from the discovery cohort. In light blue, the VUS variants of the KDR and KIT genes are indicated. Numbers in squares indicate the total number of multiple mutations detected in each sample.

Mutation analysis, which was conducted on FFPE samples of primary tumors using a next-generation sequencing (NGS) array, revealed a total of 77 single gene mutations within our study population: 26 mutations were identified in cases, while 51 were observed in controls (median mutation rates: 3.3 vs. 6.6; average mutation rates per sample: 3.7 vs. 7.3; *p* = 0.013). In Figure 1, all somatic mutations detected in the discovery cohort of our series are reported. 

Notably, the most frequently mutated gene was *TP53*, accounting for 27 pathogenic/likely pathogenic (P/LP) mutations in 13/14 (93%) patients; subsequent genes included *CDKN2A*, with a total of 9 P/LP mutations in 7/14 (50%) patients. Interestingly, these two latter genes were found to carry multiple mutations in some patients (Figure 1). We also included the variants of unknown significance (*VUS*) in *KIT* and *KDR* (though in the literature, these are mostly reported as likely benign variants); altogether, such genes were found mutated in 3 (43%) cases and 7 (100%) controls (Figure 1).

In addition to genes found pathogenically mutated in both cases and controls (though with discrepant prevalence for some of them, as for NOTCH1 and SMAD4; see Figure 1), mutations occurred in different genes among cases and controls in a large fraction of them.

Among the eight patients with multiple mutations detected in TP53 and CDKN2A genes (Figure 1), it is noteworthy that about two thirds of them (5/8; 63%) had a history of additional neoplastic diseases. Specifically, two patients had a background of non-Hodgkin’s lymphoma, one had a history of prostatic adenocarcinoma, another one had a prior renal carcinoma, and the last one had a history of chronic lymphatic leukemia. Within this same group, two patients later developed a non-melanoma skin cancer (NMSC) in other body sites during follow-up period.

**Figure 2:**
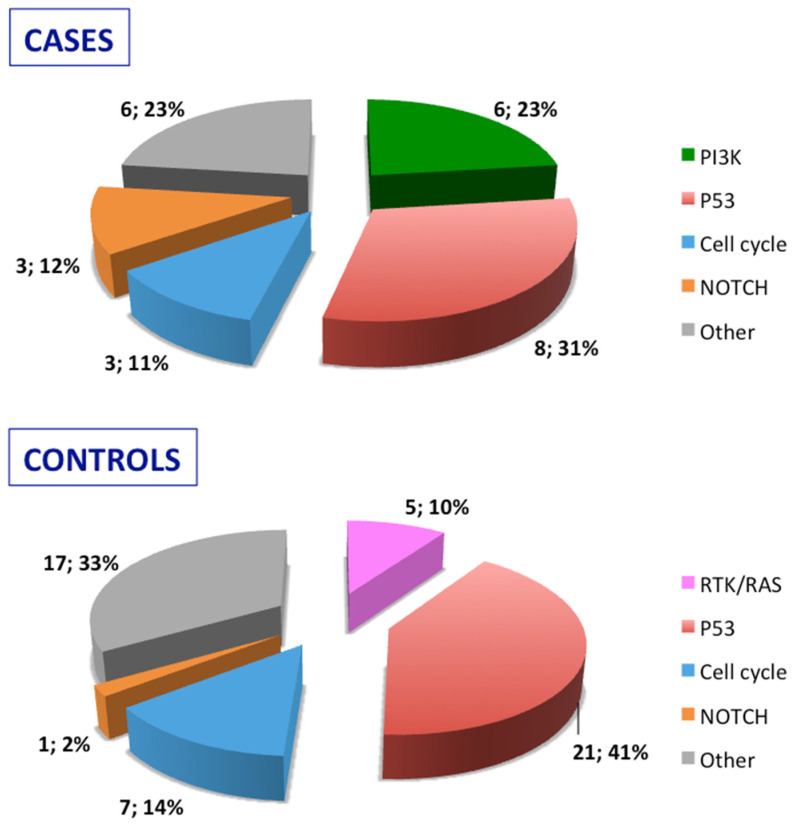
Distribution and frequencies of mutated genes in cases and controls by oncogenic pathways. Receptor Tyrosine Kinase (RTK)/RAS pathway: FGFR1, ERBB2, and FLT3; *PI3K/PTEN pathway*: PIK3CA, PTEN, and STK11; *P53-ATM pathway*: TP53 and ATM; *cell cycle pathway*: CDKN2A and RB1; *NOTCH pathway*: NOTCH1; *other genes*: SMAD4, IDH2, GNAS, EZH2, KIT, and KDR.

Stratification of the mutated genes was then performed according to the molecular pathways they belong to for both relapsed cases and relapse-free controls (Figure 2). Mutated genes were then grouped according to their role in oncogenic signaling pathways recognized in the cancer genome atlas [18]: RTK/RAS pathway, PI3K pathway, p53 pathway, NOTCH pathway, and cell cycle pathway. As detailed in Figure 2, the PIK pathway was involved in cases but not in controls, and, conversely, the RTK/RAS pathway was exclusively affected in controls.

To further confirm such a discrepancy in the mutation landscape between cases and controls in our Sardinian population, we identified a validation cohort of 12 cSCC patients presenting with the same features previously described (onset in the head or neck region and pT2cN0cM0 disease stage at diagnosis) but a shorter follow-up period (median, 2.3 years; range 2.1–2.9 years). Again, the cases were 6 patients who developed a local relapse within one year of surgical excision of the primary lesion, and the controls were 6 patients who did not relapse during the follow-up period. The vast majority of cSCCs in the validation cohort were located on the scalp (10/12; 83%), while the remaining two (17%) lesions were detected on the cheeks. The average age in the validation cohort was 77 (median 78, range 68–86); again, nearly all patients in this series were male (11/12; 92%).

**Figure 3:**
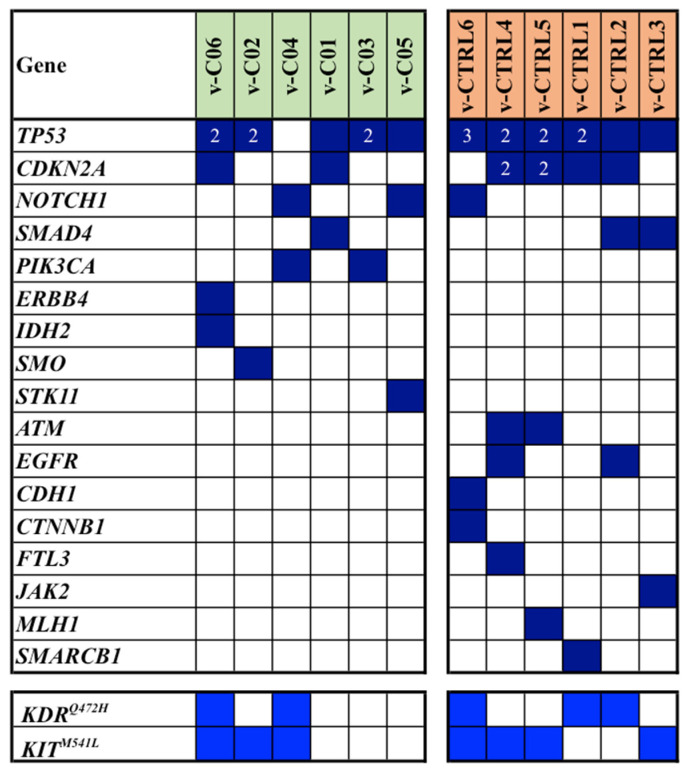
Spectrum and distribution of pathogenic/likely pathogenic mutations (dark blue squares) among cases (v-C) and controls (v-CTRL) from the validation cohort. In light blue are indicated the VUS variants of the KDR and KIT genes. Numbers in the squares indicate the total of multiple mutations detected in each sample.

As shown in Figure 3, TP53 and CDKN2A were again the most frequently mutated genes, and their mutations, along with those in NOTCH1 and SMAD4, were observed in both cases and controls of the series. Analogously to the mutation pattern found in the discovery cohort, the majority of mutated genes were differently distributed among cases and controls (Figure 3). 

Overall, NGS-based analysis revealed a total of 61 single gene mutations. Of these, 24 were identified in cases, while 37 were observed in controls (median mutation rates: 3.5 vs. 6.3; average mutation rates per sample: 4.0 vs. 6.2; *p* = 0.046). In Figure 3, all somatic mutations detected in the validation cohort of our series are reported.

## 4. Discussion

Nowadays, cutaneous squamous cell carcinoma is one of the most common malignancies encountered in our clinical practice, with a rising incidence [19]. It represents the second most common skin cancer in terms of both prevalence and mortality.

Although cSCC usually carries a favourable prognosis in its early stages, this outlook deteriorates in more advanced cases. Hence, early diagnosis and a comprehensive understanding of the most suitable treatment for each individual patient are essential. Depending on its location and clinical and pathological features [17], cSCC may present with high-risk or very high-risk profiles and can even show local invasion at the time of clinical presentation. In these scenarios, many challenges related to surgical procedures and reconstructions arise, and consideration for both functional and cosmetic implications must guide therapeutic decisions. Consequently, since advanced cSCC may impose significant risks in terms of its impact on quality of life, morbidity, mortality, and increased management costs, a systematic approach to treatment is imperative [20].

Our study falls within the field of research aimed at exploring molecular features that may potentially improve therapeutic strategies for the better management of cancer patients. In particular, this case-control study aims to examine the mutation profiles of high-risk cSCC lesions from the head and neck region in order to assess the eventual existence of a molecular signature able to confer or protect from proneness to local recurrence. In other words, the goal is to molecularly refine the prognosis evaluation of cSCC toward a more accurate support for current therapeutic strategies in the disease.

For the development of this study, considering discovery and validation cohorts, two subsets of cSCC patients were retrospectively selected: one group who encountered an early local relapse of the disease (cases) and the second group of controls who never relapsed during the follow-up period (for more than 2 and more than 4 years after surgical excision of the primary tumor in validation and discovery cohorts, respectively).

From the qualitative point of view, our findings further emphasized that TP53 and CDKN2A genes were deeply involved in cSCC pathogenesis. This is in agreement with the existing literature [8,10]. In our series, other genes commonly associated with this malignancy—such as NOTCH1-2-3, RAS, and FAT1—were observed only in a few cases or even remained unidentified (RAS and FAT1). Notably, KIT and KDR gene variants were found at higher prevalence in our series than expected, though they are not classified as pathogenic, and their functional role has not been fully established yet. 

Overall, the most interesting result from our study is represented by the statistically significant differences in the quantitative levels of mutations between cases and controls; in the latter subset of cSCC patients, a higher mutational rate is indeed associated with a lack of local recurrence in the same follow-up period. One could speculate that our evidence of a higher rate of mutations (even using a limited multigenic NGS panel) might be indicative of a tendency to have a more extensively high tumor mutation load, which in turn can make the tumors more immunogenic and thus prevent the tumor relapse (see below) [21].

Since our study population predominantly consisted of male patients (with only two female patients), no assessment of any potential molecular difference between the two sexes was feasible.

As previously described, five of the selected patients had a personal history of previous neoplastic disease, and, notably, about two-thirds of these individuals exhibited a heavy mutational load in both TP53 and CDKN2A tumor suppressor genes (see Figure 1). One plausible explanation could be that these patients may possess a peculiar impairment of the molecular mechanisms suppressing cell proliferation and thus present a constitutive predisposition to developing malignancies. Further studies with a broader range of cases are thereby imperative to support these hypotheses.

Although this study did not identify specific mutations as prognostic or therapeutic targets, an intriguing finding lies in the difference in mutation rate between the cases and the controls. Contrary to what is conventionally expected, the control group where no recurrence was observed displayed a trend towards a higher mutation burden compared to the case group. This may seem paradoxical, since it is commonly believed that disease aggressiveness correlates with higher mutation burden in the neoplastic tissue. Nonetheless, it is worth noting that tumor cells produce neoantigens recognized and targeted by the immune system [8]. To explain the discrepancy in mutations between our cases and controls, we assume that a higher number of mutations within neoplastic tissue could result in the greater exposure of potential antigens by the neoplastic cells. This, in turn, might facilitate a more effective targeting and attack by the immune system in the control group’s tumors. It is precisely this mechanism that we believe could fortify and sustain immunological defenses, potentially reducing the risk of subsequent recurrences.

In support of this hypothesis is also the evidence that neoplastic cells employ molecular mimicry as one of the primary strategies to evade the immune system and facilitate disease progression. Through molecular mimicry, these cells mimic normal cellular components, hindering effective targeting by the body’s defenses. The PD-L1/PD-1 axis plays a pivotal role in immune evasion in cancer, serving as the rationale for developing new drugs in recent years. Considering this malignancies’ behaviour and the comprehension of immune checkpoints, immunotherapy treatments have been developed and applied in several oncologic settings, often yielding profound and robust responses. Tumors with a higher tumor mutational burden are known to be more responsive to immune checkpoint inhibitors [22,23], and cSCC exhibits the highest mutational burden among all malignancies, making it a promising candidate for immunotherapy treatment [6]. To substantiate this argument, it is well documented that systemic immunosuppression, whether arising iatrogenically or through systemic disease, is a significant risk factor for cSCC development, underscoring the importance of the immune system in this tumor [8,24]. Moreover, in the context of immunosuppression, cSCC often manifests as multiple lesions and tends to be more aggressive [25,26].

Immunotherapy, especially in combination with radiotherapy, has gained popularity as a treatment option for locally advanced or metastatic cSCC. This approach has proven effective, especially for cases ineligible for curative primary surgery, showing a reduction in tumor mass in a few months and maintaining results over time [27,28]. Emerging evidence suggests that immunotherapy holds promise as a future option of care due to its tolerability and efficacy [28,29]. We believe that this efficacy could be attributed to the sustained activation of the immune system, persisting even after tumor eradication and thus reducing the risk of relapse and the development of additional cSCCs elsewhere. The first drug specifically approved by Health Canada and the U.S. Food and Drug Administration for advanced cSCC treatment is Cemiplimab [30,31]. This monoclonal antibody belongs to the class of immune checkpoint inhibitors and specifically acts as an anti–PD-1 systemic immunotherapy. It works by binding to the PD-1 receptor expressed on lymphocytes, preventing interaction with its ligands, PD-L1 and PD-L2, expressed on cancer cells. As a result, Cemiplimab demonstrates significant antitumor activity in patients with metastatic and locally advanced cSCC. Normally, when the PD-1 receptor expressed on the T cells binds with the PD-L1 ligand present on neoplastic cells, it suppresses T cells activity, inducing anergia and consequently inhibiting the immune response. Thus, cancer cells can evade immune surveillance, allowing for tumor growth. Cemiplimab, however, could overcome the lack of recognition of cancer cells by cytotoxic T cells and activate an antitumor immune response.

Tumors characterized by a high mutation burden tend to respond better to checkpoint inhibitor immunotherapy, and this heightened responsiveness is believed to be attributed, at least in part, to their increased neoantigen load and corresponding immunogenicity [32]. In a similar way, we hypothesized that neoplasms with the highest mutation rates in our study would probably elicit a more robust response from the immune system. This could potentially explain why the control group exhibited a less aggressive course during the follow-up period. Notably, none of the patients examined in our study were immune depressed, providing supports for this hypothesis. However, it is essential to acknowledge that this theory would benefit from further validation through additional studies involving a wider range of cases.

## 5. Conclusions

The results of our study are consistent with data from the existing literature, showing TP53 and CDKN2A as the most frequently mutated genes in cSCC [8,10,33].

Upon comparing mutational results found in the different patient groups, no outcomes emerged that would designate individual mutations as specific molecular targets for therapy. Surprisingly, contrary to expectations, the cases reported fewer mutations than the controls. This leads us to assume that a higher number of mutations may prompt the neoplasm to expose a greater number of antigens, making it a more accessible target for the immune system. Consequently, this could trigger a resilient and enduring immunological response, potentially preventing the occurrence of relapses over time. These findings highlight the key role of the immune system in cSCC pathogenesis and progression, emphasizing the significance of new systemic therapies that leverage the endogenous immune response.

Further studies with broader samples size are imperative to validate the formulated hypothesis and to explore its potential application in advancing new therapies.

## Data Availability

The data used and/or analyzed during the current study are available from the corresponding author upon reasonable request.
